# Relevance of blast counts for genetic subclassification in MDS

**DOI:** 10.1038/s41375-024-02484-4

**Published:** 2024-11-27

**Authors:** Sandra Huber, Torsten Haferlach, Stephan Hutter, Gregor Hoermann, Wolfgang Kern, Claudia Haferlach

**Affiliations:** MLL Munich Leukemia Laboratory, Max-Lebsche-Platz 31, 81377 Munich, Germany

**Keywords:** Genomics, Haematological diseases

## To the Editor:

We read the paper by Al Amri et al. on the independent prognostic significance of blast count in a large cohort of patients with myelodysplastic neoplasms (MDS) with great interest [1]. The study compared the MDS classifications proposed by the 5th edition of the WHO classification (WHO 2022), the International Consensus Classification (ICC), and our previous study (MLL class) with the molecular international prognostic scoring system (IPSS-M) and focused on the relevance of blast count. Both recently proposed classifications, WHO 2022 and ICC, emphasize a combination of clinicopathologic and genetic features for MDS classification [2, 3]. To overcome differences between both classifications a harmonized classification for MDS was proposed by the International Consortium for Myelodysplastic Syndromes (icMDS) [4]. We asked the provocative question: “Do we still have to count blasts?”, and suggested a blast-independent categorization of MDS stratifying patients only by genetic features [5]. Based on the genetic landscape and associations between genetic abnormalities we proposed nine genetically defined non-overlapping hierarchical subgroups: 1. bi*TP53*, 2. complex karyotype, 3. mutated *RUNX1* (*RUNX1* + ), 4. mutated *ASXL1* (*ASXL1* + ), 5. del(5q) (5q-), 6. mutated *SF3B1* (*SF3B1* + ), 7. mutated *U2AF1*, *SRSF2*, and/or *ZRSR2* (SP + ), 8. presence of at least one mutation in *DNMT3A* or *TET2* or one of other genes recurrently mutated in MDS (SP-/ ≥ 1), 9. complete absence of genetic markers defining any of the previous entities (SP-/0). While prognosis is not the main subject of disease classification as discussed below, we also used survival data as a surrogate for biology and found an impact of our classification on overall survival as independently confirmed by Al Amri et al. [1].

**Fig. 1:**
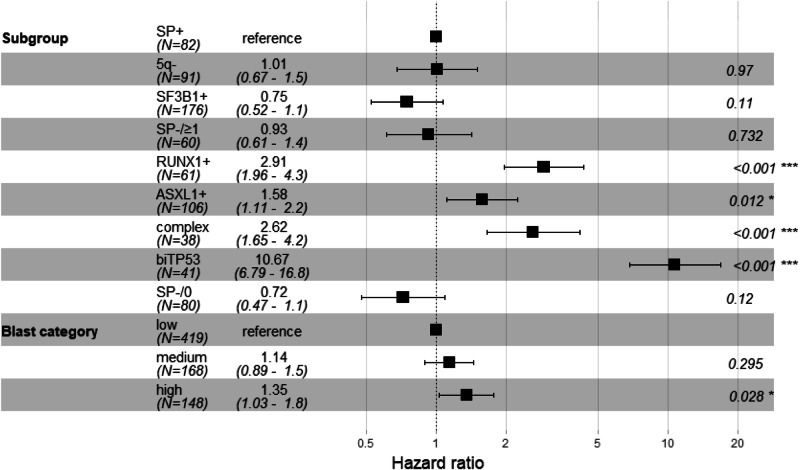
Forest plot of Cox regression model. Hazard ratios are illustrated for the nine genetically defined subgroups (upper part) and the blast categories (lower part; low: <5%, medium: 5 to <10%, high: 10 to <20%). */***statistically significant.

When specifically investigating the remaining relevance of the blast cell count, we used a Cox proportional hazards regression model to identify the impact of different variables. In univariate analyses the categories bi*TP53*, complex karyotype, *RUNX1* + , *ASXL1* + , *SF3B1* + , absence of genetic markers (SP-/0) and blast counts <5% vs ≥5% and <10% vs ≥10% were significantly associated with overall survival. Bi*TP53*, complex karyotype, *RUNX1*+ and *ASXL1*+ were poor, while *SF3B1* + , SP-/0, <5% and <10% blasts were good prognostic markers. In multivariate analyses blast count cut-offs of 5% and 10% did not show an independent significant impact on overall survival, while the categories bi*TP53*, complex karyotype, *RUNX1* + , *ASXL1* + , *SF3B1*+ did. However, the impact of the 10% blast count cutoff just did not reach statistical significance with a *p*-value of 0.063 most likely due to sample size. When using three instead of two groups for blast counts (low: <5%, medium: 5 to <10%, high: 10 to <20%) as described by Al Amri et al. [1] high blast counts retained their prognostic impact in multivariate analysis (Fig. 1). In this line, Al Amri et al. emphasize the current need for incorporating blast quantification into the classification of MDS (even when considering genetic information), as they found an independent prognostic significance of blast counts in MDS.

**Fig. 2:**
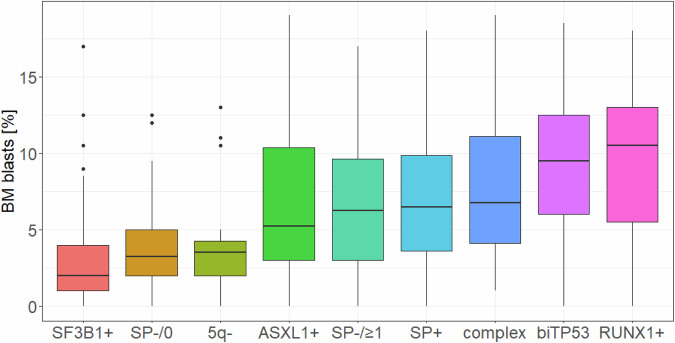
Analysis of BM blasts for the nine genetically defined subgroups depicted by boxplots showing the distribution and median of blasts. Subgroups are ordered by their median blast count. BM bone marrow.

While we do confirm in our model that the blast cell count shows some remaining prognostic impact, we should differentiate ‘classification’ from ‘prognosis’. Disease classification intends to identify patients with homogeneous biology, while prognostic models aim at grouping patients with homogeneous outcomes, irrespective of underlying biological similarities as reflected by the IPSS-M. In our study the aim was to push the classification of MDS towards a more genetically based approach believing that a more genetics-driven approach better reflects biological subgroups than a primarily morphology based classification. Differences in survival are just a poor surrogate for biological differences of the proposed genetic subgroups of MDS and can be heavily influenced by different therapies. Genetics on the other hand is able to define biological subgroups more directly which can foster development of specific treatment strategies in the area of precision medicine. In this context, we found that our genetically-defined subgroups correlate with blast counts (Fig. 2), thereby recognizing the prognostic impact of blast counts within certain subgroups. We demonstrated in our study that MDS can be separated into nine biologically distinct subgroups based on karyotype and mutation status of 21 genes. Bernard et al. followed a similar concept and characterized 16 molecular groups in MDS using information from 21 genes, 6 cytogenetic events, and LOH at the *TP53* and *TET2* loci [6]. In that model the prognostic impact of blast counts depended on the genetic subtype while it has to be acknowledged that this also depends on the number of samples per group and the respective statistical power [6].

As current MDS classifications rely on arbitrary blast cutoffs entailing challenges associated with issues of accuracy, reproducibility and inter-observer variability [7], classification based on less subjective factors is strongly aimed for. An ideal disease classification should reflect the pathobiology as accurately as possible, which is to date in the best and most available way reflected by genetics. Some remaining open questions are: the role of epigenetics; gene dosage effects; clone size; and clonal evolution over time. In addition, clinical aspects like the severity of symptoms (e.g. transfusion dependence) need to be addressed. Bernard et al. further suggested a novel paradigm where the genetic subtypes should be the overarching basis of disease classification, while blasts should designate the stage of the disease within certain genetic subgroups as also proposed by Kewan et al. [8]. This concept elegantly combines the advantages of a genetic and a clinicopathologic disease classification and for now represents a suitable modus operandi. Stratifying primarily by genetics for the underlying biology of the disease and then adding clinicopathologic variables to define the stage of the disease could be helpful to define more biologically homogeneous groups of patients and thus foster targeted drug development. Still, blast cell count rather indirectly reflects the stage of the disease and is a result of underlying biologic or genetic events that should be better characterized in the long term.

## Data Availability

The datasets generated during and/or analyzed during the current study are available from the corresponding author on reasonable request.

## References

[1] Al Amri R, Baloda V, Monaghan SA, Rosado FG, Moore EM, Rea B, et al. Validation of independent prognostic significance of blast count in a large cohort of MDS patients. Leukemia. 2024;38:2064–7. 39014198 10.1038/s41375-024-02348-x

[2] Khoury JD, Solary E, Abla O, Akkari Y, Alaggio R, Apperley JF, et al. The 5th edition of the World Health Organization Classification of Haematolymphoid Tumours: Myeloid and Histiocytic/Dendritic Neoplasms. Leukemia. 2022;36:1703–19. 35732831 10.1038/s41375-022-01613-1 PMC9252913

[3] Arber DA, Orazi A, Hasserjian RP, Borowitz MJ, Calvo KR, Kvasnicka H-M, et al. International Consensus Classification of Myeloid Neoplasms and Acute Leukemias: integrating morphologic, clinical, and genomic data. Blood. 2022;140:1200–28. 35767897 10.1182/blood.2022015850 PMC9479031

[4] Komrokji RS, Lanino L, Ball S, Bewersdorf JP, Marchetti M, Maggioni G, et al. Data-driven, harmonised classification system for myelodysplastic syndromes: a consensus paper from the International Consortium for Myelodysplastic Syndromes. Lancet Haematol. 2024. 10.1016/S2352-3026(24)00251-5. 10.1016/S2352-3026(24)00251-5 39393368

[5] Huber S, Haferlach T, Müller H, Meggendorfer M, Hutter S, Hoermann G, et al. MDS subclassification-do we still have to count blasts? Leukemia. 2023;37:942–5. 36813994 10.1038/s41375-023-01855-7 PMC10079547

[6] Bernard E, Hasserjian RP, Greenberg PL, Arango Ossa JE, Creignou M, Tuechler H, et al. Molecular taxonomy of myelodysplastic syndromes and its clinical implications. Blood. 2024;144:1617–32. 38958467 10.1182/blood.2023023727 PMC11487646

[7] Chen X, Fromm JR, Naresh KN. “Blasts” in myeloid neoplasms - how do we define blasts and how do we incorporate them into diagnostic schema moving forward? Leukemia. 2022;36:327–32. 35042955 10.1038/s41375-021-01498-6

[8] Kewan T, Durmaz A, Bahaj W, Gurnari C, Terkawi L, Awada H, et al. Molecular patterns identify distinct subclasses of myeloid neoplasia. Nat Commun. 2023;14:3136. 37253784 10.1038/s41467-023-38515-4 PMC10229666

